# The Perception of Spirituality and Its Assessment among Those with Different Health Statuses in Saudi Arabia

**DOI:** 10.3390/healthcare11142034

**Published:** 2023-07-16

**Authors:** Mohammed Alrukban, Abdulaziz Alrabiah, Faisal Alomri, Abdulaziz Alghuligah, Abdulaziz Alderaywsh, Abdulaziz Alomar, Abdulaziz Alkraida

**Affiliations:** 1 College of Medicine, King Saud University, Riyadh 11149, Saudi Arabia; 2 Family and Community Medicine Department, King Khalid University Hospital, Riyadh 11149, Saudi Arabia

**Keywords:** spiritual care, spiritual needs, Saudi Arabia, religion, spirituality

## Abstract

This study compares the perception of spirituality among individuals with different health statuses. It also describes the spiritual services and religious support in the healthcare system that are recommended by the community. A cross-sectional comparative study was conducted in Riyadh during the year 2021. A face-to-face questionnaire was used to obtain data from seriously ill inpatients (N = 132), chronically ill outpatients (N = 202), and healthy individuals (N = 283), which is an assessment tool designed by the investigators to meet the purpose of the study. This study was conducted in two tertiary hospitals and in the community. Participants were randomly selected using a stratified random sampling technique. The study was performed on 635 participants. A significant number of the participants agreed that the provision of spiritual services has a positive effect on patient condition. The rural population had a higher mean value on limiting the concept of spirituality to religious aspects. There is a clear tendency from all categories toward religious services. The majority of the participants support the provision of psychological services, especially if it is provided by specialized and expert spiritual care providers. The positive perception of participants about spiritual care has been reflected on the tendency toward providing spiritual services. The provision of spiritual care in healthcare systems is expected to be a necessity and not a luxury.

## 1. Introduction

Spirituality is an aspect of humanity that helps an individual to experience connection to oneself, to the environment, to the moment, and the significance of the sacred [1]. Spirituality refers to the quest of an individual for the meaning of purpose [1]. There are strong indicators of the importance of spiritual need, which is reflected by the increasing number of studies that investigate spirituality-based interventions [2]. Spiritual needs can be defined as a domain that contains four interconnected aspects, i.e., relationship, restfulness, purpose, and sublimity, and are affected by underlying psychological, experiential, and religious needs [3]. Spiritual needs include the need to be loved, understood, and valued and the need for hope, trust, and forgiveness; it is basically the need to find purpose in life [4].

Spirituality might aid patients in coping with illnesses and pandemics, in reducing healthcare costs, and in increasing adaptation after being diagnosed with serious disease [5]. It was observed that applying efficient spiritual care may enhance a patients’ satisfaction and have a positive effect on the quality of care [6]. Patients often find hope and meaning through spirituality [7]. Both spirituality and religion have a beneficial impact on the recovery of seriously ill patients [8].

The management of seriously ill patients is more than just caring for the person’s body [9]. Spiritual care plays a significant role in cancer patients’ management. Cancer patients usually develop more spiritual needs after discovering their illness and prefer to have spiritual support within global care [7,10]. Several studies [6,10,11] in the US found that the prevalence of spiritual needs in advanced cancer patients ranges from 44% to 91%. It is found that at least one spiritual need is indicated in 90% of cancer patients [10,12]. A large number of studies confirm that spirituality among cancer patients is beneficial in patients’ physical and functional well-being, as well as in reducing stress symptoms among cancer patients [13,14,15]. Therefore, providing spiritual care in healthcare systems is a necessity and not a luxury.

Spirituality and religion are often used interchangeably, but they possess distinct characteristics. According to some scholars, spirituality involves an individual’s personal exploration of life’s significance, while religion encompasses an organized institution with rituals and practices centered around a higher power or God [16]. Religiosity plays a vital role in patient care because it positively influences the course of illness and significantly affects the mortality rate [17,18]. Vander Weele et al. [18] reported a decreasing mortality rate in people who attend religious services several times per week compared to those who never attend any religious service. Furthermore, according to another study, health-related quality of life might be improved by engaging in religious practices, having intrinsic religious beliefs, and having religious coping methods [19,20]. Additionally, in a study of religious practices in Saudi patients with colorectal cancer, it was found that religious practices like praying, Qur’an recitation, and engaging in charity improve patients’ health and help them cope with their illness [21].

Healthcare providers implement various methods of spiritual therapeutics and support with their patients. In a study by Mamier et al. [22], it is found that the most common provided spiritual practices to the patients were “remaining with a patient after completing a task to show caring”, “Assessing patients’ spiritual beliefs pertaining to health”, “listening to patients’ stories of illness”, and “listening to patients’ spiritual concerns”. Additionally, dealing with patients would require healthcare workers to manifest non-judgmental acceptance of patients’ spiritual pain, as well as to listen attentively in order to provide their spiritual essentials [23].

In the second half of the 20th century, spirituality and religion in healthcare became insignificant in a lot of Western countries [24]. In the Netherlands, the role of spirituality and religion became almost marginal in the national healthcare system [24]. In the US, the frequency of unmet spiritual needs in cancer patients ranges from 14% to 54% [25]. Selman et al. [26] suggested that every healthcare provider that is responsible for seriously ill patients should have the basic level of proficiency in spiritual care. An integrative review by Lin et al. [27] indicates that the provision of spiritual care by health professionals has an important role in improving psycho-spiritual well-being. In a study applied on 231 physicians, 58% believed that physicians are supposed to meet patients’ spiritual concerns [28].

Furthermore, assessing spirituality is often not considered when discussing patients’ management [29]. To implement effective spiritual care, recognizing different individuals’ perspectives of spirituality is essential [30]. On the other hand, both cancer patients (84%) and healthy individuals (76%) value the provision of spiritual care in healthcare systems [9,30].

The purpose of this study is to compare the perceptions of seriously ill patients, chronically ill patients, and healthy individuals towards spirituality. It also assessed the level of spiritual services provided in the hospitals and described the psychological services and religious support in the healthcare systems that are recommended by the community. The research questions were as follows:

Research Question 1: What are the differences in the perceptions of spirituality among seriously ill patients, chronically ill patients, and healthy individuals?

Research Question 2: What is the extent of spiritual services provided in hospitals for seriously ill patients?

Research Question 3: What are the psychological services and religious support practices recommended by the community to be integrated into healthcare systems?

## 2. Materials and Methods

A quantitative cross-sectional comparative study was conducted in the city of Riyadh, Saudi Arabia, from June 2021 to October 2021. The study was conducted in an inpatient setting with those in a seriously ill condition (major organ failure, ICU COVID-19, and cancer patients), with chronically ill outpatient clinic visitors (medicine, family medicine, and surgery), and with healthy individuals. Inpatient and outpatient data were obtained from two tertiary hospitals. The data of healthy individuals were obtained by dividing Riyadh city into five sectors.

The sample size was 600, with a confidence interval of 95%, error margin of 0.04, and *p* = 0.51, according to the literature, representing the minimum number of patients recommending spiritual care [10,12,29]. A total of 635 questionnaires were collected to overcome the non-respondence rate.

Inclusion criteria were all seriously ill patients, outpatients with chronic follow-ups in the clinic, and healthy adult individuals not suffering from any chronic conditions in the city of Riyadh during the period of data collection. However, patients with severe psychological conditions, children below 15 years of age, and those with severe health conditions were excluded from the study.

Convenience sampling was employed as the method of participant recruitment. Participants were randomly selected using a stratified random sampling technique. The targeted population was stratified into three main strata according to health status as follows: inpatients with seriously ill conditions, chronically ill outpatient clinic visitors, and healthy individuals. Seriously ill and chronically ill patients were selected from all main hospitals in Riyadh. However, due to the military regulation and COVID-19 pandemic, the study was conducted in the King Khalid University Hospital (KKUH) and the King Abdullah bin Abdulaziz University Hospital (KAAUH) in Riyadh.

First, seriously ill patients were randomly selected from KKUH and KAAUH by taking random samples from patients in all seriously ill patient wards. Second, chronically ill patients were randomly selected from KKUH and KAAUH in various outpatient clinics (medicine, family medicine, and surgery). Third, healthy individuals were sampled after dividing the city of Riyadh into five sectors (North, South, East, West, and Middle). A total of 25 districts were randomly chosen from these sectors, and random samples were obtained from parks, malls, streets, restaurants, etc. All of the targeted population were eligible to participate in the study.

After approval from the KSU IRB and KAAUH IRB committees, with project number E21-6153, trained data collectors obtained the data using a face-to-face questionnaire, which is an assessment tool designed by the investigators to meet the purpose of the study. The questionnaire included various variables distributed into three themes. The first theme included demographic variables (age, gender, socioeconomic status, length of hospital stay (LOS), etc.) and health status of participants. LOS was divided into short, medium, and long stays, which were less than or equal to 5 days, 6–20 days, and more than or equal to 21 days, respectively [31,32].

Furthermore, total household monthly income was divided into low (less than or equal to SAR 9999), medium (between SAR 10,000 and 20,000), and high (more than SAR 20,000). The outcome variables were divided into two main themes. The first theme assessed the perception of seriously ill patients, chronically ill patients, and healthy individuals. The second theme described the spiritual services and religious support in the healthcare system recommended by the community. These variables were assessed using the Likert scale (1932) and the answers were rated into the following categories: 1: strongly disagree; 2: disagree; 3: natural; 4: agree; 5: strongly agree.

The questionnaire was validated in two ways. First, a pilot study was conducted on 18 subjects to validate its comprehensibility in meeting the objectives and to estimate the time needed to complete the survey. Second, the questionnaire was revised and evaluated by experts based on the study objectives.

Data were analyzed using the SPSS version 21.0 (IBM Corporation, Armonk, NY, USA) statistical software. Categorical and quantitative variables were described using descriptive statistics (frequencies, percentages, mean, and standard deviation). Bivariate analysis was conducted using one-way analysis of variance followed by a post hoc test for quantitative outcome variables, to compare the mean values in relation to the categorical study variables which have more than two options. A *p*-value of ≤0.05 was used to report the statistical significance of the results. Furthermore, the objectives of the study were explained to the participants prior to participation.

## 3. Results

### 3.1. Sociodemographic and Health Status Characteristics

The study was performed on 635 participants. A total of 617 of the participants completed the questionnaire with a 97.1% response rate. Table 1 shows that approximately 74% of respondents are male, 93.4% live in an urban area, 65.8% are married, and 86.7% are Saudi. The majority of the participants (49.8%) had a “Diploma/bachelor’s degree”. Participants were distributed into three categories, and almost 46% were healthy with a mean age of 33 years. Chronically ill patients accounted for 32.7%, with an average age of 45 years. From this, 48.5% were from King Khalid University Hospital (KKUH), 42.1% were from King Abdullah bin Abdulaziz University Hospital, and 9.4% were from other hospitals. The most common chronic conditions were diabetes (35.1%), hypertension (27.7%), heart disease (13.4%), arthritis (11.9%), and asthma (10.9%). Seriously ill patients accounted for 21.3%, with an average age of 53 years, and the majority (98.5%) were from KKUH. The most common serious illness conditions were major organ failure (49.2%), cancer (48.5%), and ICU COVID-19 patients (2.3%).

### 3.2. Participants’ Perception of Nature and Importance of Spiritual/Religious Care in Relation to Their Health Status

Table 2 shows the approximately high levels of the mean perception rates of participants toward spiritual care. The item with the highest total mean was “Spiritual/religious support plays a role in improving the patient’s condition”, with a score of 4.40. The item “Providing spiritual/religious care is important in the healthcare system” had a statistically significant difference in the mean perception of seriously ill patients compared to the healthy group, and this difference represents an increase in the mean of seriously ill patients. The item “I have a clear background about the available spiritual/religious services in the hospital” had the lowest total mean of perception, with a value of 2.76. This item also showed a high statistically significant difference between seriously ill patients and healthy individuals (*p* < 0.001), but this difference represents a decrease in the mean perception of the seriously ill group (2.34).

### 3.3. Level of Spiritual/Religious Care Provided to Seriously Ill Patients in Hospitals

Among 131 responses from seriously ill patients, two-thirds did not receive spiritual/religious care during hospitalization (Table 3). On the other hand, the hospitalized patients who received spiritual/religious care showed medium levels of satisfaction (3.56–3.95).

### 3.4. Participants Preferences towards Religious Services in the Healthcare System in Relation to Their Health Status

The majority of the participants support the availability of religious services, and all the religious services’ means were above 3.64 (Table 4). It was observed that “Providing interactive religious lectures” gained the lowest acceptance mean of 3.64. The following four services scored a high level of recommendation with no statistical differences: “Provide a dress that preserves the personal hygiene and privacy of the patient” (4.32), “Providing wudu alternatives for patients that cannot use water“ (4.27), “Providing Suitable place for congregational prayer” (4.22), and “Providing Azan sound at prayers times” (4.18). The acceptance for most of the services by healthy individuals was statistically significant compared to seriously and chronically ill patients. The seriously ill patients displayed less importance in addressing the following services compared to the healthy group: “Providing Islamic recitation over patient”, “Answering religious questions”, “Providing Qibla Direction Sign and Directing beds toward Qibla”, and “Providing Prophetic Medicine and its nutritional supplement”, and all were statistically significant (Table 4). Two services also showed statistically significant differences between the healthy and chronically ill patients, which were “Providing Prophetic Medicine and its nutritional supplement” (*p* < 0.001) and “Providing devices to listen to Quran” (*p* < 0.05), with the means in the healthy group higher than those of the chronically ill patients (Table 4).

### 3.5. Participants’ Preferences towards Psychological Services in the Healthcare System in Relation to Their Health Status

Regarding psychological services, the majority of the participants support providing all suggested services with a total mean above 4.22. The service with the highest total mean was “Ensuring a calm and stable environment inside patients’ room” (4.47). The lowest mean of seriously ill patients among the services recommended was 3.87 for the item “Helping to get involved in volunteer work, including support group”. This item also had a statistically significant difference between seriously ill patients and healthy individuals. The item “Hearing patient concern and complaints” (*p* < 0.05) was also statistically significant, which represents an increase in the mean of seriously ill patients (4.34). The item “Continuing the spiritual care after discharging from the hospital” showed a statistically significant difference between seriously ill patients and the other two categories (healthy, chronically ill patients), which represents a low mean for seriously ill patients (3.72) compared to the others (Table 5).

### 3.6. Participants Preferred Spiritual Care Providers

The participants showed high levels of acceptability towards the appropriateness of different spiritual care providers. The most preferred spiritual care providers were palliative care specialists (526, 85.3%) and then the person with the experience to provide spiritual care regardless of his specialty (522, 84.6%), while the least preferred spiritual care providers were doctors (402, 65.2%) and nurses (344, 55.8%). There were no statistically significant differences between sample categories.

## 4. Discussion

The concept of “spiritual need” is not often discussed in the healthcare system worldwide. This study compared the perception of communities with different health statuses toward spirituality and described the different spiritual services recommended by them.

From the findings, a prominent level of perception toward spiritual care existed among all participants and there were no significant differences between demographic variables. Furthermore, it revealed the strong support from all participants towards providing various religious and psychological spiritual services.

The majority of the population had a positive perception toward spiritual support and recommended the provision of spiritual care in the healthcare system. Similar findings could be observed in other studies. A study by Selman et al. [26] showed that patients understand the positive effect of spiritual care [13,14,15]. In Saudi Arabia, despite the patients’ belief of the importance of religious-based interventions during life-threatening situations, the perception of Saudi Muslims about the relationship between religiosity, spirituality, and health is low [33,34].

Unfortunately, the majority of the participants did not receive spiritual care and had no idea about spiritual care facilities in hospitals. Similar findings were observed in a study conducted in the US, where 75% of cancer patients confirmed that their spiritual needs are not investigated and not supported [35]. According to Rushton et al. [36], several studies have shown that healthcare providers failed to address patient spiritual needs in hospitals, particularly from health professionals. Furthermore, a nine-country focus group study reported a wide-spread carelessness by healthcare providers in providing spiritual care [26]. They justified this carelessness as not having enough time to address these issues with patients [36].

From the findings, the majority of the population consider religious services as a part of spiritual care. Sastra et al. [37] agreed with this and demonstrated the importance of considering religious needs for Indonesian Muslim cancer patients. Similarly, another study by Astro et al. [38] showed that 50% of patients appreciated it when their religious belief or spiritual needs were addressed by their doctors. However, the high religiosity level of the community may have an impact on religious services’ addressing [20].

According to participants, the application of spiritual care should not be limited to religious issues but should also consider other aspects of spiritual support. This finding reflects the high corroboration towards providing psychological services. Applying spiritual care gives hope and meaning to patients and helps them to cope with their illness [7]. Several studies observed that providing spiritual care to seriously ill patients would improve their quality of life and mental health, as well as reduce the use of aggressive treatments [39,40,41]. However, even non-religious persons have a group of spiritual needs, especially the need of inner peace and giving attention, which supports the result of the item that states “The application of spiritual care revolves around the religious aspect only” [42].

Both religious and psychological services are important in the enhancement of effective spiritual care. Spirituality and religiosity significantly contribute to psychosocial adjustment towards cancer and its treatments [19]. Cruz et al. [20] used the health-related quality of life (HRQoL) scale on 168 Saudi patients undergoing hemodialysis and found a high psychological/spiritual score, with a noticeable importance of religiosity and spiritual coping (SC). They recommended the integration of religiosity in healthcare services for better healthcare services [20].

Interestingly, the study shows a significant tendency of the rural population to limit the concept of spirituality to the religious aspect only compared to the urban population. This finding supports the role of residency in defining the relationship between religiosity and spiritual care. Several studies have shown variations in spiritual services’ delivery between rural and urban areas according to Carey et al. [43]. A study conducted in the United States has shown no significant differences in the desire of rural and urban patients to receive spiritual and religious assessment [44].

All healthcare workers can be a source of spiritual care delivery according to the study findings. However, participants demonstrate preferences for the provision spiritual support by specialized and expert spiritual care providers. Spiritual support is expected to be a role of all healthcare providers. It was reported that physicians can effectively provide spiritual care, and religious physicians tend to consider spiritual support with their patients [45].

Furthermore, nurses could have a significant role in spiritual support if they overcome the challenges. Healthcare providers can face many challenges in the healthcare system, including the pressures of clinical duties, lack of training, and the absence of credentials [46]. Poor provision of spiritual support to patients is caused mainly by the absence of training to healthcare workers [47]. Providing spiritual care training would fill the gap and surge patients’ satisfaction with provided care [47]. 

It was found that the length of hospital stay has no significant effect on the need for spiritual care. Patients with both short and long hospital stays admit a positive perception. On the other hand, according to Plöderl et al. [48], only a small and non-significant association existed between the level of religiosity/spirituality and the length of hospital stay.

This study has potential limitations. The data collectors encountered difficulty in conducting the questionnaire with seriously ill patients due to their critical status. Also, some of the demographic characteristics are not representative of the population, which should be taken into consideration in future research.

## 5. Conclusions

The study findings concluded that the perception of spiritual support tends to be positive among the majority of the participants. Furthermore, a large portion of participants suggested that providing religious and psychological spiritual services can have a positive impact on the patient’s condition. To provide comprehensive healthcare services, a spiritual care provider advised that patients’ spiritual needs should be addressed and spiritual support provided accordingly. It is expected that providing spiritual care by the right person and using the right method will improve patients’ satisfaction and could be cost-effective. Future studies should focus on measuring the spiritual needs of different categories of seriously ill patients. Additionally, healthcare providers should be trained on how to implement spiritual care.

## Figures and Tables

**Table 1:** Distribution of socio-demographic characteristics of healthy individuals, chronically ill, and seriously ill patients.

Variable	Response	Healthy N (%)	Chronically Ill N (%)	Seriously Ill N (%)
Age	15–34	167 (59)	53 (26.2)	22 (16.7)
	35–54	108 (38.2)	84 (41.6)	39 (29.5)
	55–84	8 (2.8)	65 (32.2)	71 (53.8)
Gender	Male	210 (74.2)	146 (72.3)	101 (76.5)
	Female	73 (25.8)	56 (27.7)	31 (23.5)
Education level	Uneducated	2 (0.7)	5 (2.5)	7 (5.3)
	Elementary	3 (1.1)	10 (5.0)	16 (12.1)
	Intermediate	11 (3.9)	14 (6.9)	13 (9.8)
	High School	78 (27.6)	49 (24.3)	31 (23.5)
	Diploma/Bachelor	155 (54.8)	90 (44.6)	62 (47.0)
	Higher Education	34 (12.0)	34 (16.8)	3 (2.3)
Marital status	Married	155 (54.8)	155 (76.7)	96 (72.7)
	Single	114 (40.3)	38 (18.8)	24 (18.2)
	Divorced/Widow	14 (4.9)	9 (4.5)	12 (9.1)
Residency	Urban	269 (95.1)	187 (92.6)	120 (90.9)
	Governorate/rural and village	14 (4.9)	15 (7.4)	12 (9.1)
Occupational status	Employed	211 (74.6)	102 (50.5)	36 (27.3)
	Unemployed	21 (7.4)	25 (12.4)	32 (24.2)
	Freelancer	12 (4.2)	8 (4.0)	10 (7.6)
	Retired	11 (3.9)	51 (25.2)	47 (35.6)
	Student	28 (9.9)	16 (7.9)	7 (5.3)
Total household monthly income	Low	172 (60.8)	99 (49.0)	76 (57.6)
	Medium	87 (30.7)	83 (41.1)	45 (34.1)
	High	24 (8.5)	20 (9.9)	11 (8.3)
Nationality	Saudi	221 (78.1)	190 (94.1)	124 (93.9)
	Non-Saudi	62 (21.9)	12 (5.9)	8 (6.1)
Length of hospital stay	Short	-	-	32 (24.2)
	Medium	-	-	53 (40.2)
	Long	-	-	47 (35.6)

**Table 2:** Comparison of mean values of participants’ perception of nature and importance of spiritual/religious care in relation to their health status.

Variable	Healthy Mean (*SD)	Chronically Ill Mean (*SD)	Seriously Ill Mean (*SD)	Total Mean	*p*-Value
Providing spiritual/religious care is important in the health care system.	4.23 (0.7958)	4.28 (0.9858)	4.50 (0.7044)	4.31	0.008
Spiritual/religious support plays a role in improving the patient’s condition.	4.31 (0.7423)	4.45 (0.7983)	4.47 (0.8423)	4.40	0.068
The application of spiritual care revolves around the religious aspect only.	2.99 (1.1093)	3.06 (1.3365)	2.85 (1.4041)	3.00	0.313
I have a clear background about the available spiritual/religious services in the hospital.	2.98 (1.1834)	2.73 (1.3036)	2.34 (1.3076)	2.76	<0.0001

*SD: standard deviation.

**Table 3:** Mean values of spiritual/religious care provided to seriously ill patients in hospitals.

Variable	Response	Seriously Ill N (%)
Did you receive spiritual/religious care during your hospitalization?	No	87 (66.4)
	Yes	44 (33.6)
I am satisfied about the level of spiritual/religious care.	Strongly Disagree	2 (4.5)
	Disagree	4 (9.1)
	Neutral	5 (11.3)
	Agree	8 (18.2)
	Strongly Agree	7 (15.9)
	Average of Satisfaction	3.91
My stay in the hospital helped to enhance my understanding of the concept of spiritual/religious support.	Strongly Disagree	5 (11.4)
	Disagree	8 (18.2)
	Neutral	7 (15.9)
	Agree	10 (22.7)
	Strongly Agree	14 (31.8)
	Average of Satisfaction	3.56
I feel comfortable when discussing my spiritual/religious issues with a spiritual/religious care specialist.	Strongly Disagree	2 (4.5)
	Disagree	3 (6.8)
	Neutral	7 (15.9)
	Agree	15 (34.1)
	Strongly Agree	17 (38.7)
	Average of Satisfaction	3.95

**Table 4:** Comparison of mean values of participants’ preferences towards religious services in the healthcare system in relation to their health status.

Variable	Healthy Mean (*SD)	Chronically Ill Mean (*SD)	Seriously Ill Mean (*SD)	Total Mean	*p*-Value
Religious Services					
Providing a Suitable place for Congregational prayer.	4.29 (0.76)	4.21 (1.00)	4.08 (1.01)	4.22	0.082
Providing a suitable place for praying in the patients’ rooms.	4.05 (0.94)	3.91 (1.08)	3.86 (1.04)	3.96	0.135
Providing Qibla Direction Sign and directing beds toward Qibla.	4.26 (0.80)	4.11 (0.94)	3.80 (1.20)	4.11	<0.0001
Providing Azan sound at prayers times.	4.19 (0.90)	4.14 (0.94)	4.24 (0.84)	4.18	0.619
Providing wudu alternatives for patients that cannot use water.	4.27 (0.79)	4.27 (0.84)	4.28 (0.87)	4.27	0.982
Provide a dress that preserves the personal hygiene and privacy of the patient.	4.329 (0.71)	4.34 (0.68)	4.28 (0.87)	4.32	0.710
Providing interactive religious lectures.	3.79 (1.03)	3.57 (1.14)	3.43 (1.38)	3.64	0.006
Provide an audio-visual religious booklet.	3.95 (0.91)	3.86 (0.95)	3.75 (1.20)	3.88	0.137
Providing Islamic recitation over patient.	3.96 (0.96)	3.84 (1.08)	3.68 (1.30)	3.86	0.048
Providing devices to listen to Quran.	4.16 (0.77)	3.96 (0.99)	3.97 (1.10)	4.05	0.034
Answering religious questions [fatwas].	4.01 (0.91)	3.83 (1.15)	3.74 (1.32)	3.89	0.036
Providing Zamzam Water for patients.	4.10 (0.91)	3.95 (1.16)	4.00 (1.26)	4.03	0.292
Providing Prophetic Medicine and its nutritional supplement [Ex: Honey, Nigella sativa].	4.00 (0.97)	3.70 (1.17)	3.56 (1.36)	3.80	<0.0001
Helping with writing a will.	3.90 (0.98)	3.80 (1.17)	3.50 (1.37)	3.78	0.004
Setting up a facility in hospital to provide a Spiritual/Religious service.	4.13 (0.85)	4.14 (0.90)	3.87 (1.25)	4.08	0.023

*SD: standard deviation.

**Table 5:** Comparison of mean values of participants’ preferences towards psychological services in the healthcare system in relation to their health status.

Variable	Healthy Mean (*SD)	Chronically Ill Mean (*SD)	Seriously Ill Mean (*SD)	Total Mean	*p*-Value
Psychological Services					
Helping to meditate, reflect, and find a purpose in life.	4.12 (0.84)	4.20 (0.99)	4.27 (1.07)	4.18	0.317
Ensuring a calm and stable environment inside patients’ room.	4.41 (0.67)	4.50 (0.72)	4.55 (0.73)	4.47	0.149
Hearing patient concerns and complaints.	4.12 (0.89)	4.32 (0.90)	4.34 (1.03)	4.23	0.027
Helping to accept the disease as well as create positive thoughts about prognosis.	4.28 (0.81)	4.41 (0.83)	4.47 (0.93)	4.36	0.059
Regulating flexible visiting times in line with the patient’s interest and increasing their communication with the family.	4.23 (0.86)	4.40 (0.92)	4.18 (1.17)	4.27	0.073
Provide someone to support during the stay in the hospital [such as: the presence of an escort].	4.28 (0.78)	4.43 (0.75)	4.43 (0.91)	4.36	0.076
Helping to get involved in support group including volunteer work.	3.99 (1.01)	3.87 (1.29)	3.64 (1.49)	3.87	0.026
Continuing the spiritual care after discharging from the hospital.	4.04 (1.00)	4.12 (1.15)	3.72 (1.42)	4.00	0.006

*SD: standard deviation.

## Data Availability

The data presented in this study are available on request from the corresponding author.

## References

[1] Puchalski C., Betty F. (2010). Making Health Care Whole: Integrating Spirituality into Patient Care.

[2] Gonçalves J.P.d.B., Lucchetti G., Menezes P.R., Vallada H. (2017). Complementary Religious and Spiritual Interventions in Physical Health and Quality of Life: A Systematic Review of Randomized Controlled Clinical Trials. PLoS ONE.

[3] Riklikienė O., Tomkevičiūtė J., Spirgienė L., Valiulienė Ž., Büssing A. (2020). Spiritual Needs and Their Association with Indicators of Quality of Life among Non-Terminally Ill Cancer Patients: Cross-Sectional Survey. Eur. J. Oncol. Nurs..

[4] Jackson D., Doyle C., Capon H., Pringle E. (2016). Spirituality, Spiritual Need, and Spiritual Care in Aged Care: What the Literature Says. J. Relig. Spirit. Aging.

[5] Del Castillo F.A. (2020). Health, Spirituality and COVID-19: Themes and Insights. J. Public Health.

[6] Astrow A.B., Wexler A., Texeira K., He M.K., Sulmasy D.P. (2007). Is Failure to Meet Spiritual Needs Associated with Cancer Patients’ Perceptions of Quality of Care and Their Satisfaction with Care?. J. Clin. Oncol..

[7] Puchalski C.M. (2012). Spirituality in the Cancer Trajectory. Ann. Oncol..

[8] Monareng L.V. (2012). Spiritual Nursing Care: A Concept Analysis. Curationis.

[9] Ripamonti C.I., Giuntoli F., Gonella S., Miccinesi G. (2018). Spiritual Care in Cancer Patients: A Need or an Option?. Curr. Opin. Oncol..

[10] Pearce M.J., Coan A.D., Herndon J.E., Koenig H.G., Abernethy A.P. (2012). Unmet Spiritual Care Needs Impact Emotional and Spiritual Well-Being in Advanced Cancer Patients. Support. Care Cancer.

[11] Hui D., de la Cruz M., Thorney S., Parsons H.A., Delgado-Guay M., Bruera E. (2011). The Frequency and Correlates of Spiritual Distress among Patients with Advanced Cancer Admitted to an Acute Palliative Care Unit. Am. J. Hosp. Palliat. Med..

[12] Höcker A., Krüll A., Koch U., Mehnert A. (2014). Exploring Spiritual Needs and Their Associated Factors in an Urban Sample of Early and Advanced Cancer Patients. Eur. J. Cancer Care.

[13] Shahmoradi N., Kandiah M., Loh S.P. (2012). Quality of Life and Functional Status in Patients with Advanced Cancer Admitted to Hospice Home Care in Malaysia: A Cross-sectional Study. Eur. J. Cancer Care.

[14] Kim Y., Seidlitz L. (2002). Spirituality Moderates the Effect of Stress on Emotional and Physical Adjustment. Pers. Individ. Dif..

[15] Rawdin B., Evans C., Rabow M.W. (2013). The Relationships among Hope, Pain, Psychological Distress, and Spiritual Well-Being in Oncology Outpatients. J. Palliat. Med..

[16] Arrey A.E., Bilsen J., Lacor P., Deschepper R. (2016). Spirituality/Religiosity: A Cultural and Psychological Resource among Sub-Saharan African Migrant Women with HIV/AIDS in Belgium. PLoS ONE.

[17] Mishra S.K., Togneri E., Tripathi B., Trikamji B. (2017). Spirituality and Religiosity and Its Role in Health and Diseases. J. Relig. Health.

[18] VanderWeele T.J. (2017). Religious Communities and Human Flourishing. Curr. Dir. Psychol. Sci..

[19] Weaver A.J., Flannelly K.J. (2004). The Role of Religion/Spirituality for Cancer Patients and Their Caregivers. South. Med. J..

[20] Cruz J.P., Colet P.C., Alquwez N., Inocian E.P., Al-Otaibi R.S., Islam S.M.S. (2017). Influence of Religiosity and Spiritual Coping on Health-related Quality of Life in Saudi Haemodialysis Patients. Hemodial. Int..

[21] Shaheen Al Ahwal M., Al Zaben F., Sehlo M.G., Khalifa D.A., Koenig H.G. (2015). Religious Beliefs, Practices, and Health in Colorectal Cancer Patients in Saudi Arabia. Psycho-Oncology.

[22] Mamier I., Taylor E.J., Winslow B.W. (2019). Nurse Spiritual Care: Prevalence and Correlates. West. J. Nurs. Res..

[23] Ferrell B.R., Handzo G., Picchi T., Puchalski C., Rosa W.E. (2020). The Urgency of Spiritual Care: COVID-19 and the Critical Need for Whole-Person Palliation. J. Pain. Symptom Manag..

[24] Van de Geer J., Veeger N., Groot M., Zock H., Leget C., Prins J., Vissers K. (2018). Multidisciplinary Training on Spiritual Care for Patients in Palliative Care Trajectories Improves the Attitudes and Competencies of Hospital Medical Staff: Results of a Quasi-Experimental Study. Am. J. Hosp. Palliat. Med..

[25] Harrison J.D., Young J.M., Price M.A., Butow P.N., Solomon M.J. (2009). What Are the Unmet Supportive Care Needs of People with Cancer? A Systematic Review. Support. Care Cancer.

[26] Selman L.E., Brighton L.J., Sinclair S., Karvinen I., Egan R., Speck P., Powell R.A., Deskur-Smielecka E., Glajchen M., Adler S. (2018). Patients’ and Caregivers’ Needs, Experiences, Preferences and Research Priorities in Spiritual Care: A Focus Group Study across Nine Countries. Palliat. Med..

[27] Lin H., Bauer-Wu S.M. (2003). Psycho-spiritual Well-being in Patients with Advanced Cancer: An Integrative Review of the Literature. J. Adv. Nurs..

[28] Ellis M.R., Vinson D.C., Ewigman B., Columbia M. (1999). Addressing Spiritual Concerns of Patients Family Physicians’ Attitudes and Practices. J. Fam. Pract..

[29] Büssing A., Recchia D.R., Koenig H., Baumann K., Frick E. (2018). Factor Structure of the Spiritual Needs Questionnaire (SpNQ) in Persons with Chronic Diseases, Elderly and Healthy Individuals. Religions.

[30] Fitch M.I., Bartlett R. (2019). Patient Perspectives about Spirituality and Spiritual Care. Asia Pac. J. Oncol. Nurs..

[31] Damiani G., Pinnarelli L., Sommella L., Vena V., Magrini P., Ricciardi W. (2011). The Short Stay Unit as a New Option for Hospitals: A Review of the Scientific Literature. Med. Sci. Monit..

[32] Venturini F., de Moura E.C., Bastos P.A., Martins L.C., Fragoso Y.D. (2018). Profile and Costs Involved in Long-Term Compulsory Hospitalization of Psychiatric Patients. Braz. J. Psychiatry.

[33] Jazieh A.R., Al Sudairy R., Abulkhair O., Alaskar A., Al Safi F., Sheblaq N., Young S., Issa M., Tamim H. (2012). Use of Complementary and Alternative Medicine by Patients with Cancer in Saudi Arabia. J. Altern. Complement. Med..

[34] Al Zaben F., Khalifa D.A., Sehlo M.G., Al Shohaib S., Binzaqr S.A., Badreg A.M., Alsaadi R.A., Koenig H.G. (2015). Religious Involvement and Health in Dialysis Patients in Saudi Arabia. J. Relig. Health.

[35] Balboni T.A., Vanderwerker L.C., Block S.D., Paulk M.E., Lathan C.S., Peteet J.R., Prigerson H.G. (2007). Religiousness and Spiritual Support among Advanced Cancer Patients and Associations with End-of-Life Treatment Preferences and Quality of Life. J. Clin. Oncol..

[36] Rushton L. (2014). What Are the Barriers to Spiritual Care in a Hospital Setting?. Br. J. Nurs..

[37] Sastra L., Büssing A., Chen C.-H., Yen M., Lin E.C.-L. (2021). Spiritual Needs and Influencing Factors of Indonesian Muslims with Cancer During Hospitalization. J. Transcult. Nurs..

[38] Astrow A.B., Kwok G., Sharma R.K., Fromer N., Sulmasy D.P. (2018). Spiritual Needs and Perception of Quality of Care and Satisfaction with Care in Hematology/Medical Oncology Patients: A Multicultural Assessment. J. Pain. Symptom Manag..

[39] Balboni T., Balboni M., Paulk M.E., Phelps A., Wright A., Peteet J., Block S., Lathan C., Vanderweele T., Prigerson H. (2011). Support of Cancer Patients’ Spiritual Needs and Associations with Medical Care Costs at the End of Life. Cancer.

[40] Moreira-Almeida A., Neto F.L., Koenig H.G. (2006). Religiousness and Mental Health: A Review. Braz. J. Psychiatry.

[41] Balboni T.A., Balboni M., Enzinger A.C., Gallivan K., Paulk M.E., Wright A., Steinhauser K., VanderWeele T.J., Prigerson H.G. (2013). Provision of Spiritual Support to Patients with Advanced Cancer by Religious Communities and Associations with Medical Care at the End of Life. JAMA Intern. Med..

[42] Büssing A., Balzat H.-J., Heusser P. (2010). Spiritual Needs of Patients with Chronic Pain Diseases and Cancer-Validation of the Spiritual Needs Questionnaire. Eur. J. Med. Res..

[43] Carey L.B., Hennequin C., Krikheli L., O’Brien A., Sanchez E., Marsden C.R. (2016). Rural Health and Spiritual Care Development: A Review of Programs across Rural Victoria, Australia. J. Relig. Health.

[44] Fuchs J.R., Fuchs J.W., Hauser J.M., Coors M.E. (2021). Patient Desire for Spiritual Assessment Is Unmet in Urban and Rural Primary Care Settings. BMC Health Serv. Res..

[45] VandeCreek L., Grossoehme D.H., Ragsdale J.R., McHenry C.L., Thurston C. (2007). Attention to Spiritual/Religious Concerns in Pediatric Practice: What Clinical Situations? What Educational Preparation?. Chaplain. Today.

[46] VandeCreek L. (2010). Defining and Advocating for Spiritual Care in the Hospital. J. Pastor. Care Couns..

[47] Balboni M.J., Sullivan A., Amobi A., Phelps A.C., Gorman D.P., Zollfrank A., Peteet J.R., Prigerson H.G., VanderWeele T.J., Balboni T.A. (2013). Why Is Spiritual Care Infrequent at the End of Life? Spiritual Care Perceptions among Patients, Nurses, and Physicians and the Role of Training. J. Clin. Oncol..

[48] Plöderl M., Kunrath S., Fartacek C. (2020). God Bless You? The Association of Religion and Spirituality with Reduction of Suicide Ideation and Length of Hospital Stay among Psychiatric Patients at Risk for Suicide. Suicide Life Threat. Behav..

